# Editors’ message June 2022

**DOI:** 10.1016/j.ajpc.2022.100351

**Published:** 2022-05-17

**Authors:** Nathan D. Wong, Erin D. Michos

**Affiliations:** a Division of Cardiology, University of California, Irvine, United States; b Division of Cardiology, Johns Hopkins University School of Medicine, United States

This issue of the *American Journal of Preventive Cardiology* (AJPC) marks our 10th issue which we are proud to present to you all. The success of the journal represents the tireless efforts of our associate editors and greater editorial board and the many reviewers which we will be forever grateful to. This month we in particular wish to thank Dr. Koos Admiraal who recently departed Elsevier as our publisher and was instrumental in his efforts to help us launch the AJPC and to provide guidance and help in our application last year to successfully obtain Pub Med and Scopus indexing. Since our first meeting in Summer 2019 at the European Society of Cardiology in Paris to propose the idea of the AJPC, he provided the greatest support to launch and sustain the journal. We now welcome our new Elsevier publisher, Diana Goetz who will help us take the journal into the future!

How well is the AJPC growing and getting exposure worldwide? We are happy to announce that the number of submissions grew from 108 to 149 from 2020 to 2021. While the great majority of the submissions were from the United States, we also had submissions from China, Finland, France, India, Iran, Japan, Sweden, and the United Arab Emirates. We particularly welcome submissions internationally and continue to encourage those from overseas to contact us if they are interested in being a reviewer and possibly editorial board members for the journal. Moreover, the current (2022) average time from submission to first decision is 4.8 weeks, to acceptance 11.1 weeks, and to first on-line publication 11.5 weeks. Very impressive, in particular, is the growth in usage of the journal – in 2020 there were 49,109 total downloads which increased to 163,330 in 2021. For the first 3 months of this year, there were 57,930 downloads, a 93% increase over the same period last year.

Our June issue features a number of key articles of great value to the preventive cardiology community. Two ASPC clinical practice statements are featured in this issue, one on evidence-based nutritional modifications to reduce atherosclerotic cardiovascular disease (ASCVD) risk and another on strategies for ASCVD risk assessment. Following is a commentary on the need for increasing virtual cardiac rehabilitation availability and then state-of-the-art reviews, one being the annual Ten Things to Know about Cardiovascular Risk Factors and the other on evaluation of blood lipids throughout a woman's lifecycle. The issue is rounded out by original research contributions on an update on lipid treatment and goal attainment in US adults, effectiveness of community-based interventions, patient experiences with familial hypercholesterolemia, cascade testing for lipoprotein(a), and on area deprivation index and anticoagulation among atrial fibrillation patients.

Finally, we wish to thank our outgoing ASPC President Peter P. Toth, MD, PhD for his tireless efforts and accomplishments too many to be named here in this short message, taking ASPC to greater heights during the past two years. We also welcome Martha Gulati, MD, MS as our incoming ASPC president who will undoubtedly lead our organization to even greater heights. We hope you will consider attending our upcoming ASPC Congress on CVD Prevention to be held July 29–31, 2022 in Louisville, Kentucky. A great program has been planned and is featured on our website (along with registration details) https://www.aspconline.org/2022congress/. Thank you for your readership and continuing support of our *AJPC* journal, which continues to grow in impact.

## Declaration of Competing Interest

The authors declare that they have no known competing financial interests or personal relationships that could have appeared to influence the work reported in this paper.

